# Supplementary material for: High-content imaging-based pooled CRISPR screens in mammalian cells
Source: J Cell Biol. 2021 Jan 19;220(2):e202008158. doi: 10.1083/jcb.202008158 (PMC7821101; doi:10.1083/jcb.202008158)
Supplement: Data S1 — describes the FACS gating strategy used in the screens. [file JCB_202008158_DataS1.pdf]

Supplementary file 1

FACS gating strategy of the screen

1. Cell gate and single cell gate

For all FACS experiments, forward versus side scatter (FSC-A vs. SSC-A) gating was used to identify cells of interest based on size and granularity; forward scatter width (FSC-W) vs. forward scatter area (FSC-A) density plot was used to exclude doublets as shown below.

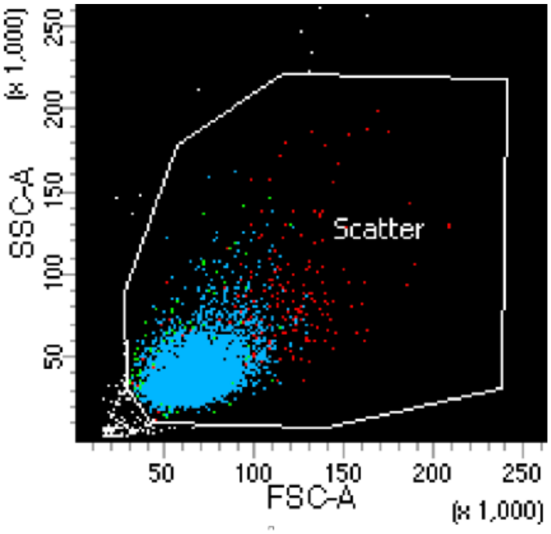

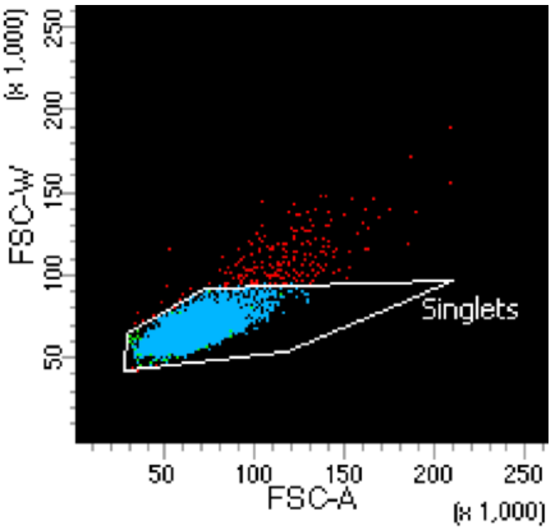

2. sgRNA gate (gate for sgRNA infected cells)

BFP (Pacific Blue-A) vs. GFP (FITC-A) density plot was used to gate H2B-mGFP cells successfully receiving sgRNAs (high BFP high GFP group). For single activation experiment (single activation mIFP proof-of-principle screen and nuclear size screen), unanalyzed samples were collected using sgRNA gate (P2 shown below).

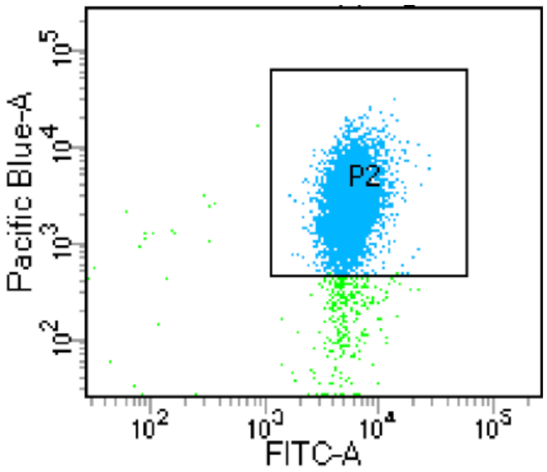

3. mCherry gate (gate for different intensity mCherry cells)

mCherry (PE-A) vs. GFP (FITC-A) density plot was used to gate for photo-activated cells. Cells activated with different photo-activation times were used to determine corresponding gates (Examples shown below). Cells in dual-activation experiments were collected with mCherry gates of corresponding photo-activation time.

0 s

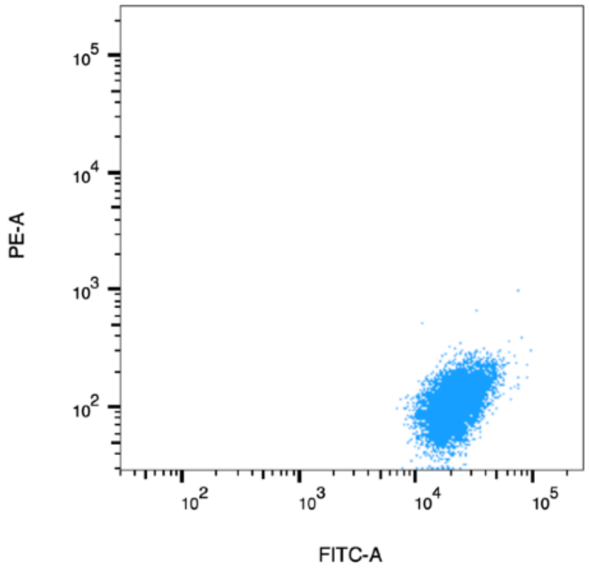

200 ms

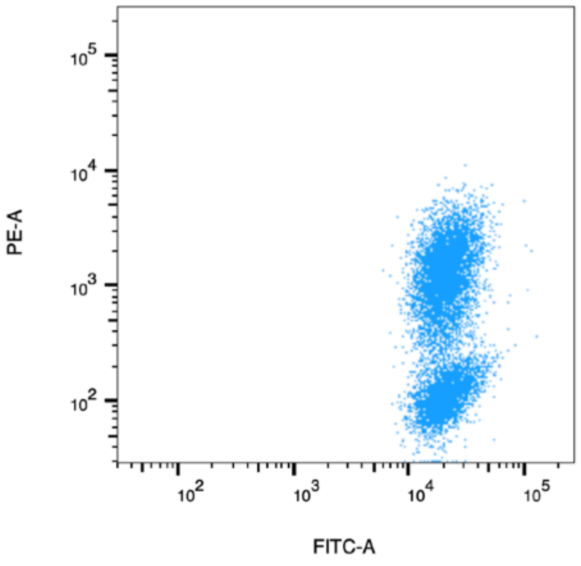

2 s

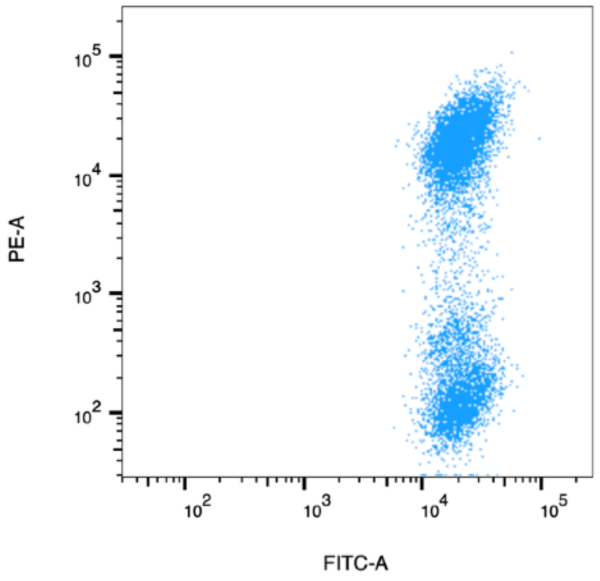

For example, in dual-activation mIFP proof-of-principle screening experiments, three distinct populations were separately sorted based on FITC-A/PE-A:

P5: positively sorted sample (2 s photo-activation)

P4: negatively sorted sample (100 ms photo-activation)
P3: unanalyzed sample (0 s photo-activation)

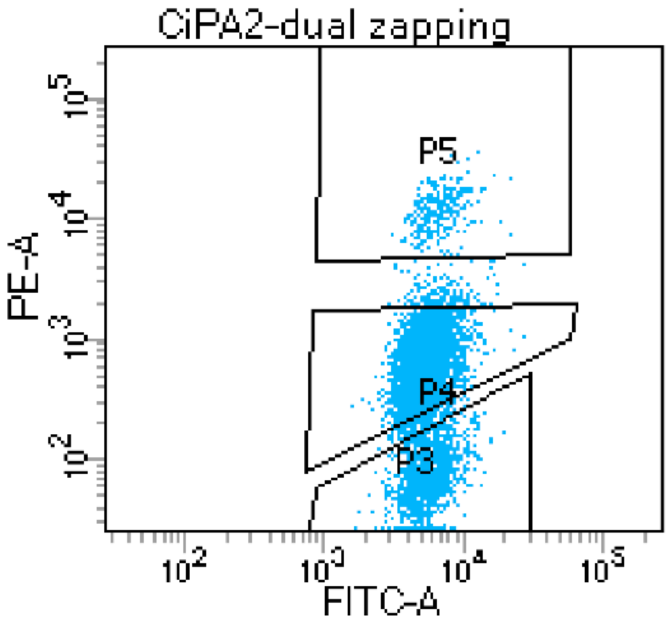

Note: laser and filters used for different channel

Channel	Laser	Filter	Measured Fluorophore
**Pacific Blue**	Violet 407 nm	450/50	BFP
**FITC**	Blue 488 nm	530/30 - 502LP	GFP
**PE**	Green 561 nm	582/15	mCherry
**APC-Cy5.5**	Red 633 nm	710/50 - 685LP	mIFP
