# Supplementary material for: High-content imaging-based pooled CRISPR screens in mammalian cells
Source: J Cell Biol. 2021 Jan 19;220(2):e202008158. doi: 10.1083/jcb.202008158 (PMC7821101; doi:10.1083/jcb.202008158)
Supplement: Data S2 — describes detailed image processing methods. [file JCB_202008158_DataS2.pdf]

Supplementary file 2

Image processing

All image processing code was written in Java and used ImageJ functions to execute image processing steps. Image processing routines were plugins to the Micro-Manager Auto-PhotoConverter plugin (unless indicated), and source code can be found at https://github.com/nicost/mnfinder branch mm2-gamma (https://doi.org/10.5281/zenodo.4274219).

1. Flatfielding and background subtraction

Background images were collected using a camera exposure of 0 ms, no illumination, and by blocking light from reaching the camera. One hundred background images were averaged. For each channel, flat field images were generated by acquiring 1000 images of cells, each slightly displaced from the other, median projection, and smoothing by Gaussian filtering. Background was subtracted, and the image was normalized so that the average of all pixels in the flat field image was 1.0.

All images acquired by the microscope were then flat field corrected by pixel wise subtraction of the background image, and division by the flat field image. This was carried out using the Micro-Manager Flat-Field Correction plugin.

2. Well edge detection

The plastic of the 96-well imaging plates is fluorescent and clearly visible in images taken at the edge of an imaging well, interfering with automated thresholding of images. To illustrate the problem, example images of automated and manual thresholding of the same image (mIFP channel) are shown below. To detect wells, the image of the user-specified channel was thresholded (threshold value was determined using the “Huang dark” ImageJ method), filled (using “Close” and “Fill Holes” commands, and expanded by a user-specified number of pixels. The detected objects were considered to be a well wall if their mean intensity was

higher than a user defined value. Well edges can either be excluded from further analysis, or images containing well edges can be skipped entirely.

Autothresholding

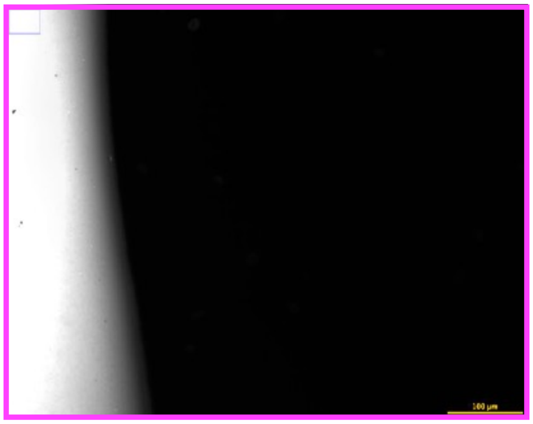

Manual thresholding

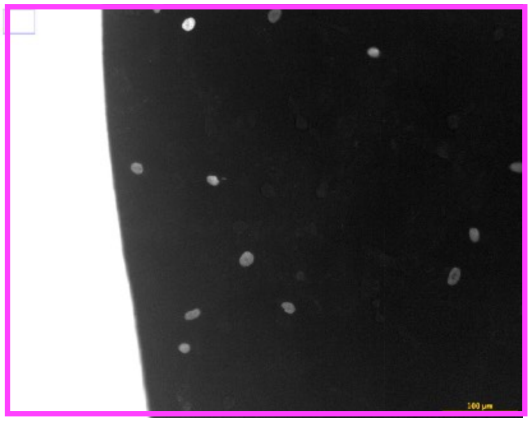

3. Image pre-processing

Even though images were background subtracted and flat field corrected, results were improved preprocessing using the ImageJ rolling ball background removal code (size 5, sliding) and the Smooth command.

Before

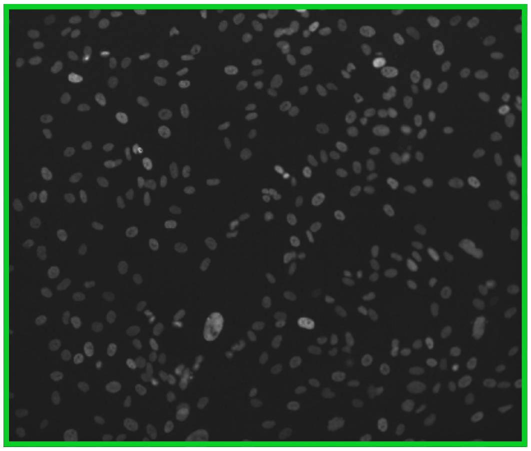

After

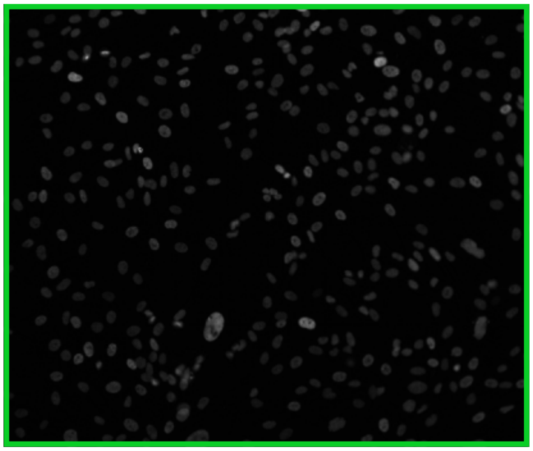

4. Initial nuclei mask generation

Threshold values for each image were calculated using the “Otsu” method and used to segment the image. Sequential application of the “Fill Holes” and “Watershed” methods resulted in binary nuclei masks.

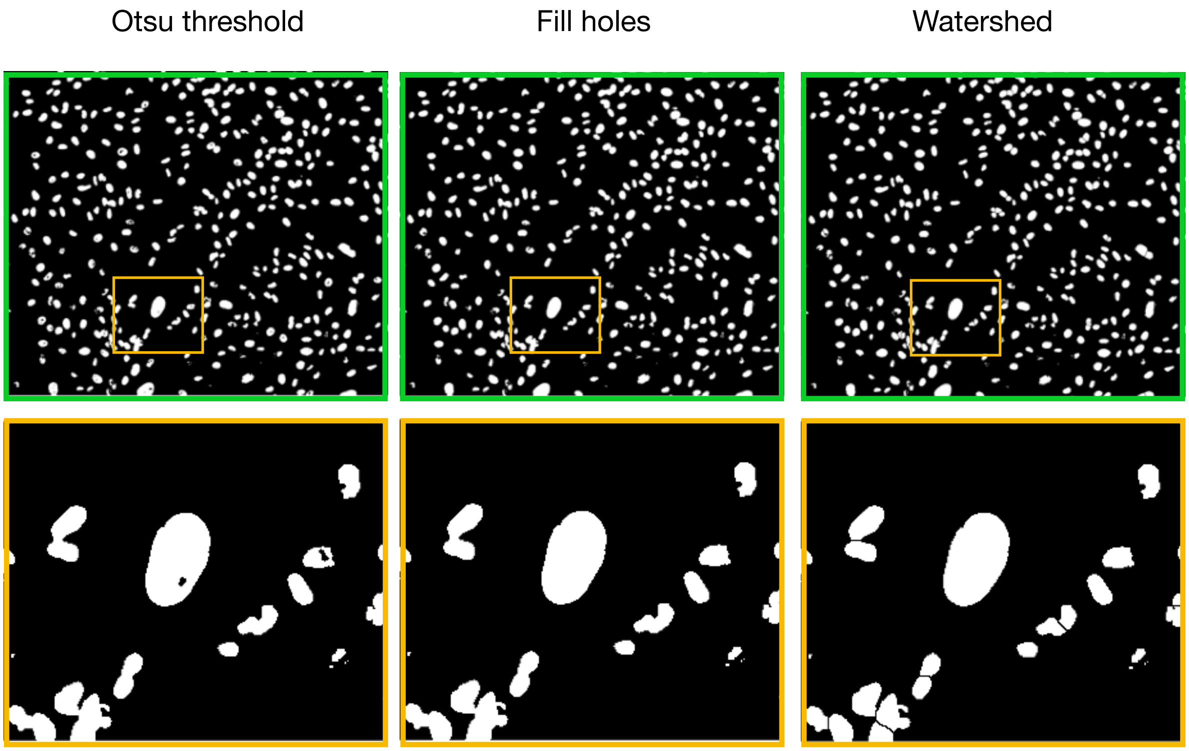

5. Nuclei filters to generate final nuclei mask

Nuclei particles within initial nuclei mask has to be filtered in order to exclude clustered cells or cells out of focus *etc.* Normal range of the following parameters were determined with control cells and nuclei particles out of range were discarded.

I Nuclei location

filter out nuclei at on the boarder of the field of view

II Nuclei size

filter out nuclei with extreme large size; these are generally clustered cells which failed to be separated from auto-thresholding

III Mean intensity of nuclei

filter out nuclei with extreme high or low intensity; these are generally dead cells, or mitotic cells which are generally out of focus

IV Standard deviation of nuclei intensity

filter out nuclei with extreme high standard deviation; these are generally not a single nucleus due to cell clustering

V Nuclei shape (measured by circularity)

filter out low and high circularity nuclei; both of these could result from cell clustering

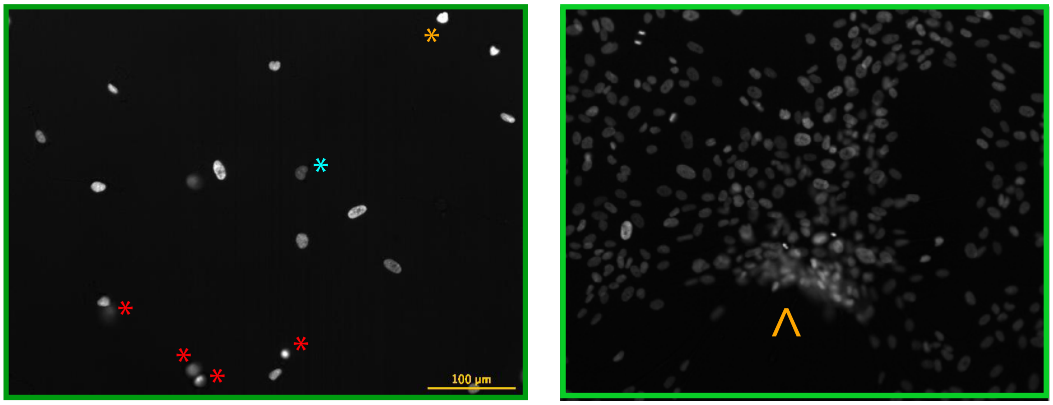

*	(red asterisk)	out of focus cells
*	(orange asterisk)	cells sitting on the boarder
*	(blue asterisk)	cells with low fluorescent intensity
^	(orange arrow)	clustered cells

6. Phenotype identification and generation of final conversion mask

I mIFP proof-of-principle screen

Measure intensity of mIFP channel of identified nuclei from previous steps. Cutoff was pre-determined with mIFP positive and negative cells.

II Nuclear size screen

Measure nuclei size of identified nuclei from previous steps. Cutoff was pre-determined with control cells.
