# Supplementary material for: High-content imaging-based pooled CRISPR screens in mammalian cells
Source: J Cell Biol. 2021 Jan 19;220(2):e202008158. doi: 10.1083/jcb.202008158 (PMC7821101; doi:10.1083/jcb.202008158)
Supplement: Data S3 — describes hits identified in both replicates of the nuclear size screen. [file JCB_202008158_DataS3.pdf]

Description of Shared Hits (summary from genecards.org, SGD and cited literature)

	Aliases	Summaries
**TICRR**	TOPBP1 Interacting Checkpoint And Replication Regulator; Treslin	Regulator of DNA replication and S/M and G2/M checkpoints. Regulates the triggering of DNA replication initiation via its interaction with TOPBP1 by participating in CDK2-mediated loading of CDC45L onto replication origins. Required for the transition from pre-replication complex (pre-RC) to pre-initiation complex (pre-IC). Required to prevent mitotic entry after treatment with ionizing radiation.
**TOP2A**	DNA Topoisomerase II Alpha	Control of topological states of DNA by transient breakage and subsequent rejoining of DNA strands. Topoisomerase II makes double-strand breaks. Essential during mitosis and meiosis for proper segregation of daughter chromosomes.
**RAD51**	RAD51 Recombinase	Plays an important role in homologous strand exchange, a key step in DNA repair through homologous recombination (HR) (PubMed:28575658). Binds to single and double-stranded DNA and exhibits DNA-dependent ATPase activity. Catalyzes the recognition of homology and strand exchange between homologous DNA partners to form a joint molecule between a processed DNA break and the repair template. Binds to single-stranded DNA in an ATP-dependent manner to form nucleoprotein filaments which are essential for the homology search and strand exchange (PubMed:26681308). Part of a PALB2-scaffolded HR complex containing BRCA2 and RAD51C and which is thought to play a role in DNA repair by HR. Plays a role in regulating mitochondrial DNA copy number under conditions of oxidative stress in the presence of RAD51C and XRCC3. Also involved in interstrand cross-link repair (PubMed:26253028).
**INCENP**	Inner Centromere Protein	Component of the chromosomal passenger complex (CPC), a complex that acts as a key regulator of mitosis. The CPC complex has essential functions at the centromere in ensuring correct chromosome alignment and segregation and is required for chromatin-induced microtubule stabilization and spindle assembly. Acts as a scaffold regulating CPC localization and activity. The C-terminus associates with AURKB or AURKC, the N-terminus associated with BIRC5/survivin and CDCA8/borealin tethers the CPC to the inner centromere, and the microtubule binding activity within the central SAH domain directs AURKB/C toward substrates near microtubules (PubMed:15316025, PubMed:12925766, PubMed:27332895). The flexibility of the SAH domain is proposed to allow AURKB/C to follow substrates on dynamic microtubules while ensuring CPC docking to static chromatin (By similarity). Activates AURKB and AURKC (PubMed:27332895). Required for localization of CBX5 to mitotic centromeres (PubMed:21346195). Controls the kinetochore localization of BUB1 (PubMed:16760428).
**AURKB**	Aurora Kinase B	Serine/threonine-protein kinase component of the chromosomal passenger complex (CPC), a complex that acts as a key regulator of mitosis. The CPC complex has essential functions at the centromere in ensuring correct chromosome alignment and segregation and is required for chromatin-induced microtubule stabilization and spindle assembly. Involved in the bipolar attachment of spindle microtubules to kinetochores and is a key regulator for the onset of cytokinesis during mitosis. Required for central/midzone spindle assembly and cleavage furrow formation. Key component of the cytokinesis checkpoint, a process required to delay abscission to prevent both premature resolution of intercellular chromosome bridges and accumulation of DNA damage: phosphorylates CHMP4C, leading to retain abscission-competent VPS4 (VPS4A and/or VPS4B) at the midbody ring until abscission checkpoint signaling is terminated at late cytokinesis (PubMed:22422861, PubMed:24814515). AURKB phosphorylates the CPC complex subunits BIRC5/survivin, CDCA8/borealin and INCENP. Phosphorylation of INCENP leads to increased AURKB activity. Other known AURKB substrates involved in centromeric functions and mitosis are CENPA, DES/desmin, GPAF, KIF2C, NSUN2, RACGAP1, SEPTIN1, VIM/vimentin, HASPIN, and histone H3. A positive feedback loop involving HASPIN and AURKB contributes to localization of CPC to centromeres. Phosphorylation of VIM controls vimentin filament segregation in cytokinetic process, whereas histone H3 is phosphorylated at 'Ser-10' and 'Ser-28' during mitosis (H3S10ph and H3S28ph, respectively). A positive feedback between HASPIN and AURKB contributes to CPC localization. AURKB is also required for kinetochore localization of BUB1 and SGO1. Phosphorylation of p53/TP53 negatively regulates its transcriptional activity. Key regulator of active promoters in resting B- and T-lymphocytes: acts by mediating phosphorylation of H3S28ph at active promoters in resting B-cells, inhibiting RNF2/RING1B-mediated ubiquitination of histone H2A and enhancing binding and activity of the USP16 deubiquitinase at transcribed genes.
**CASP8AP2**	Caspase 8 Associated Protein 2	Participates in TNF-alpha-induced blockade of glucocorticoid receptor (GR) transactivation at the nuclear receptor coactivator level, upstream and independently of NF-kappa-B. Suppresses both NCOA2- and NCOA3-induced enhancement of GR transactivation. Involved in TNF-alpha-induced activation of NF-kappa-B via a TRAF2-dependent pathway. Acts as a downstream mediator for CASP8-induced activation of NF-kappa-B. Required for the activation of CASP8 in FAS-mediated apoptosis. Required for histone gene transcription and progression through S phase.
**CDCA8**	Cell Division Cycle Associated 8; Borealin	Component of the chromosomal passenger complex (CPC), a complex that acts as a key regulator of mitosis. The CPC complex has essential functions at the centromere in ensuring correct chromosome alignment and segregation and is required for chromatin-induced microtubule stabilization and spindle assembly. Major effector of the TTK kinase in the control of attachment-error-correction and chromosome alignment.
**KIF11**	Kinesin Family Member 11; Eg5	Motor protein required for establishing a bipolar spindle during mitosis (PubMed:19001501). Required in non-mitotic cells for transport of secretory proteins from the Golgi complex to the cell surface (PubMed:23857769).
**NUP62**	Nucleoporin 62	Essential component of the nuclear pore complex (PubMed:1915414). The N-terminal is probably involved in nucleocytoplasmic transport (PubMed:1915414). The C-terminal is involved in protein-protein interaction probably via coiled-coil formation, promotes its association with centrosomes and may function in anchorage of p62 to the pore complex (PubMed:1915414, PubMed:24107630). Plays a role in mitotic cell cycle progression by regulating centrosome segregation, centriole maturation and spindle orientation (PubMed:24107630). It might be involved in protein recruitment to the centrosome after nuclear breakdown (PubMed:24107630).

	Aliases	Summaries
**SPDL1**	Spindle Apparatus Coiled-Coil Protein 1; Spindly	Required for the localization of dynein and dynactin to the mitotic kintochore. Dynein is believed to control the initial lateral interaction between the kinetochore and spindle microtubules and to facilitate the subsequent formation of end-on kinetochore-microtubule attachments mediated by the NDC80 complex. Also required for correct spindle orientation. Does not appear to be required for the removal of spindle assembly checkpoint (SAC) proteins from the kinetochore upon bipolar spindle attachment (PubMed:17576797, PubMed:19468067). Acts as an adapter protein linking the dynein motor complex to various cargos and converts dynein from a non-processive to a highly processive motor in the presence of dynactin. Facilitates the interaction between dynein and dynactin and activates dynein processivity (the ability to move along a microtubule for a long distance without falling off the track) (PubMed:25035494).
**SKA1**	Spindle And Kinetochore Associated Complex Subunit 1	Component of the SKA1 complex, a microtubule-binding subcomplex of the outer kinetochore that is essential for proper chromosome segregation (PubMed:17093495, PubMed:19289083, PubMed:23085020). Required for timely anaphase onset during mitosis, when chromosomes undergo bipolar attachment on spindle microtubules leading to silencing of the spindle checkpoint (PubMed:17093495). The SKA1 complex is a direct component of the kinetochore-microtubule interface and directly associates with microtubules as oligomeric assemblies (PubMed:19289083). The complex facilitates the processive movement of microspheres along a microtubule in a depolymerization-coupled manner (PubMed:19289083). Affinity for microtubules is synergistically enhanced in the presence of the ndc-80 complex and may allow the ndc-80 complex to track depolymerizing microtubules (PubMed:23085020). In the complex, it mediates the interaction with microtubules (PubMed:19289083, PubMed:23085020).
**DNA2**	DNA Replication Helicase/Nuclease 2	Key enzyme involved in DNA replication and DNA repair in nucleus and mitochondrion. Involved in Okazaki fragments processing by cleaving long flaps that escape FEN1: flaps that are longer than 27 nucleotides are coated by replication protein A complex (RPA), leading to recruit DNA2 which cleaves the flap until it is too short to bind RPA and becomes a substrate for FEN1. Also involved in 5'-end resection of DNA during double-strand break (DSB) repair: recruited by BLM and mediates the cleavage of 5'-ssDNA, while the 3'-ssDNA cleavage is prevented by the presence of RPA. Also involved in DNA replication checkpoint independently of Okazaki fragments processing. Possesses different enzymatic activities, such as single-stranded DNA (ssDNA)-dependent ATPase, 5'-3' helicase and endonuclease activities. While the ATPase and endonuclease activities are well-defined and play a key role in Okazaki fragments processing and DSB repair, the 5'-3' DNA helicase activity is subject to debate. According to various reports, the helicase activity is weak and its function remains largely unclear. Helicase activity may promote the motion of DNA2 on the flap, helping the nuclease function.
**FBXO5**	F-Box Protein 5; Emi1	Regulator of APC activity during mitotic and meiotic cell cycle (PubMed:17485488, PubMed:17234884, PubMed:17875940, PubMed:23708001, PubMed:23708605, PubMed:16921029). During mitotic cell cycle plays a role as both substrate and inhibitor of APC-FZR1 complex (PubMed:29875408, PubMed:17485488, PubMed:17234884, PubMed:17875940, PubMed:23708001, PubMed:23708605, PubMed:16921029). During G1 phase, plays a role as substrate of APC-FZR1 complex E3 ligase (PubMed:29875408). Then switches as an inhibitor of APC-FZR1 complex during S and G2 leading to cell-cycle commitment (PubMed:29875408). As APC inhibitor, prevents the degradation of APC substrates at multiple levels: by interacting with APC and blocking access of APC substrates to the D-box coreceptor, formed by FZR1 and ANAPC10; by suppressing ubiquitin ligation and chain elongation by APC by preventing the UBE2C and UBE2S activities (PubMed:23708605, PubMed:23708001, PubMed:16921029). Plays a role in genome integrity preservation by coordinating DNA replication with mitosis through APC inhibition in interphase to stabilize CCNA2 and GMNN in order to promote mitosis and prevent rereplication and DNA damage-induced cellular senescence (PubMed:17234884, PubMed:17485488, PubMed:17875940). During oocyte maturation, plays a role in meiosis through inactivation of APC-FZR1 complex. Inhibits APC through RPS6KA2 interaction that increases FBXO5 affiniy for CDC20 leading to the metaphase arrest of the second meiotic division before fertilization (By similarity). Controls entry into the first meiotic division through inactivation of APC-FZR1 complex (By similarity). Promotes migration and osteogenic differentiation of mesenchymal stem cells (PubMed:29850565).
**KRI1**	KRI1 Homolog	Studied mostly in S. cerevisiae and C. elegans. In S. cerevisiae, Kri1p is an essential nucleolar protein required for 40S ribosome biogenesis; associate with snR30; physically and functionally interacts with Krr1p. KRI1 is also involved in cell death regulation in C. elegans (Ito, S., Greiss, S., Gartner, A. & Derry, W. B. Cell-nonautonomous regulation of C. elegans germ cell death by kri-1. *Curr Biol* **20**, 333-338, doi:10.1016/j.cub.2009.12.032 (2010)).
