# Supplementary material for: High-content imaging-based pooled CRISPR screens in mammalian cells
Source: J Cell Biol. 2021 Jan 19;220(2):e202008158. doi: 10.1083/jcb.202008158 (PMC7821101; doi:10.1083/jcb.202008158)
Supplement: Data S4 — lists plasmid sequences used in this study. [file JCB_202008158_DataS4.pdf]

Supplementary file 4

Plasmid sequences

PA-mCherry
cgataccgtcgacctcgagggaattaattcgagctcggtacctttaagaccaatgacttacaaggcagctgtagatcttagccactt
tttaaaagaaaaggggggactggaagggctaattcactcccaacgaagacaagatctgctttttgcttgtactgggtctctctggtt
agaccagatctgagcctgggagctctctggctaactagggaacccactgcttaagcctcaataaagcttgccttgagtgcttcaag
tagtgtgtgcccgtctgttgtgtgactctggtaactagagatccctcagacccttttagtcagtgtggaaaatctctagcagcatctag
aattaattccgtgtattctatagtgtcacctaaatcgtatgtgtatgatacataaggttatgtattaattgtagccgcgttctaacgacaat
atgtacaagcctaattgtgtagcatctggcttactgaagcagaccctatcatctctctcgtaaactgccgtcagagtcggtttggttg
gacgaaccttctgagtttctggtaacgccgtcccgcacccggaaatggtcagcgaaccaatcagcagggtcatcgctagccag
atcctctacgccggacgcatcgtggccggcatcaccggcgccacaggtgcggttgctggcgcctatatcgccgacatcaccga
tggggaagatcgggctcgccacttcgggctcatgagcgcttgtttcggcgtgggtatggtggcaggccccgtggccgggggact
gttgggcgccatctccttgcatgcaccattccttgcggcggcggtgctcaacggcctcaacctactactgggctgcttcctaatgc
aggagtcgcataagggagagcgtcgaatggtgcactctcagtacaatctagctctgatgccgcatagttaagccagccccgaca
cccgccaacacccgctgacgcgccctgacgggcttgtctgctcccggcatccgcttacagacaagctgtgaccgtctccggga
gctgcatgtgtcagaggttttcaccgtcatcaccgaaacgcgcgagacgaaagggcctcgtgatacgcctatttttataggttaatg
tcatgataataatggtttcttagacgtcaggtggcacttttcggggaaatgtgcgcggaacccctatttgtttatttttctaaatacattc
aaatatgtatccgctcatgagacaataaccctgataaatgcttcaataatattgaaaaaggaagagtatgagtattcaacatttccgt
gtcgcccttattcccttttttgcggcattttgccttcctgtttttgctcacccagaaacgctggtgaaagtaaaagatgctgaagatcag
ttgggtgcacgagtgggttacatcgaactggatctcaacagcggtaagatccttgagagttttcgccccgaagaacgttttccaatg
atgagcacttttaaagttctgctatgtggcgcggtattatcccgtattgacgccgggcaagagcaactcggtcgccgcatacactat
tctcagaatgacttggttgagtactcaccagtcacagaaaagcatcttacggatggcatgacagtaagagaattatgcagtgctgc
cataaccatgagtgataacactgcggccaacttacttctgacaacgatcggaggaccgaaggagctaaccgcttttttgcacaac
atgggggatcatgtaactcgccttgatcgttgggaaccggagctgaatgaagccataccaaacgacgagcgtgacaccacgat
gcctgtagcaatggcaacaacgttgcgcaaactattaactggcgaactacttactctagcttcccggcaacaattaatagactgga
tggaggcggataaagttgcaggaccacttctgcgctcggcccttccggctggctggtttattgctgataaatctggagccggtgag
cgtgggtctcgcggtatcattgcagcactggggccagatggtaagccctcccgtatcgtagttatctacacgacggggagtcagg
caactatggatgaacgaaatagacagatcgctgagataggtgcctcactgattaagcattggtaactgtcagaccaagtttactcat
atatactttagattgatttaaaacttcatttttaatttaaaaggatctaggtgaagatcctttttgataatctcatgaccaaaatcccttaac
gtgagttttcgttccactgagcgtcagaccccgtagaaaagatcaaaggatcttcttgagatcctttttttctgcgcgtaatctgctgct
tgcaaacaaaaaaaccaccgctaccagcggtggtttgtttgccggatcaagagctaccaactctttttccgaaggtaactggcttc
agcagagcgcagataccaaatactgtccttctagtgtagccgtagttaggccaccacttcaagaactctgtagcaccgcctacat
acctcgctctgctaatcctgttaccagtggctgctgccagtggcgataagtcgtgtcttaccgggttggactcaagacgatagttac
cggataaggcgcagcggtcgggctgaacggggggttcgtgcacacagcccagcttggagcgaacgacctacaccgaactga
gatacctacagcgtgagctatgagaaagcgccacgcttcccgaagggagaaaggcggacaggtatccggtaagcggcaggg
tcggaacaggagagcgcacgagggagcttccagggggaaacgcctggtatctttatagtcctgtcgggtttcgccacctctgact
tgagcgtcgatttttgtgatgctcgtcaggggggcggagcctatggaaaaacgccagcaacgcggcctttttacggttcctggcct
tttgctggccttttgctcacatgttctttcctgcgttatcccctgattctgtggataaccgtattaccgcctttgagtgagctgataccgct
cgccgcagccgaacgaccgagcgcagcgagtcagtgagcgaggaagcggaagagcgcccaatacgcaaaccgcctctcc
ccgcgcgttggccgattcattaatgcagctgtggaatgtgtgtcagttagggtgtggaaagtccccaggctccccagcaggcaga
agtatgcaaagcatgcatctcaattagtcagcaaccaggtgtggaaagtccccaggctccccagcaggcagaagtatgcaaag
catgcatctcaattagtcagcaaccatagtcccgcccctaactccgcccatcccgcccctaactccgcccagttccgcccattct
ccgccccatggctgactaattttttttatttatgcagaggccgaggccgcctcggcctctgagctattccagaagtagtgaggaggc
ttttttggaggcctaggcttttgcaaaaagcttggacacaagacaggcttgcgagatatgtttgagaataccactttatcccgcgtca
gggagaggcagtgcgtaaaaagacgcggactcatgtgaaatactggtttttagtgcgccagatctctataatctcgcgcaacctat
tttcccctcgaacactttttaagccgtagataaacaggctgggacacttcacatgagcgaaaaatacatcgtcacctgggacatgtt
gcagatccatgcacgtaaactcgcaagccgactgatgccttctgaacaatggaaaggcattattgccgtaagccgtggcggtctg
taccgggtgcgttactggcgcgtgaactgggtattcgtcatgtcgataccgtttgtatttccagctacgatcacgacaaccagcgcg
agcttaaagtgctgaaacgcgcagaaggcgatggcgaaggcttcatcgttattgatgacctggtggataccggtggtactgcggt
tgcgattcgtgaaatgtatccaaaagcgcactttgtcaccatcttcgcaaaaccggctggtcgtccgctggttgatgactatgttgtt
gatatcccgcaagatacctggattgaacagccgtgggatatgggcgtcgtattcgtcccgccaatctccggtcgctaatcttttcaa

cgcctggcactgccgggcgttgttctttttaacttcaggcgggttacaatagtttccagtaagtattctggaggctgcatccatgaca
caggcaaacctgagcgaaaccctgttcaaaccccgctttaaacatcctgaaacctcgacgctagtccgccgctttaatcacggc
gcacaaccgcctgtgcagtcggcccttgatggtaaaaccatccctcactggtatcgcatgattaaccgtctgatgtggatctggcg
cggcattgacccacgcgaaatcctcgacgtccaggcacgtattgtgatgagcgatgccgaacgtaccgacgatgatttatacgat
acggtgattggctaccgtggcggcaactggatttatgagtgggccccggatctttgtgaaggaaccttacttctgtggtgtgacata
attggacaaactacctacagagatttaaagctctaaggtaaatataaaatttttaagtgtataatgtgttaaactactgattctaattgttt
gtgtattttagattccaacctatggaactgatgaatgggagcagtggtggaatgcctttaatgaggaaaacctgttttgctcagaaga
aatgccatctagtgatgatgaggctactgctgactctcaacattctactcctccaaaaaagaagagaaaggtagaagaccccaag
gactttccttcagaattgctaagtttttttgagtcatgctgtgtttagtaatagaactcttgcttgctttgctatttacaccacaaaggaaaa
agctgcactgctatacaagaaaattatggaaaaatattctgtaacctttataagtaggcataacagttataatcataacatactgtttttt
cttactccacacaggcatagagtgtctgctattaataactatgctcaaaaattgtgtacctttagctttttaatttgtaaaggggttaata
aggaatatttgatgtatagtgccttgactagagatcataatcagccataccacatttgtagaggttttacttgctttaaaaaacctccca
cacctccccctgaacctgaaacataaaatgaatgcaattgttgttgttaacttgtttattgcagcttataatggttacaaataaagcaat
agcatcacaaatttcacaaataaagcatttttttcactgcattctagttgtggtttgtccaaactcatcaatgtatcttatcatgtctggat
caactggataactcaagctaaccaaaatcatcccaaacttcccaccccataccctattaccactgccaattacctagtggtttcattt
actctaaacctgtgattcctctgaattattttcattttaaagaaattgtatttgttaaatatgtactacaaacttagtagttggaagggctaa
ttcactcccaaagaagacaagatatccttgatctgtggatctaccacacacaaggctacttccctgattagcagaactacacacca
gggccaggggtcagatatccactgacctttggatggtgctacaagctagtaccagttgagccagataaggtagaagaggccaat
aaaggagagaacaccagcttgttacaccctgtgagcctgcatgggatggatgacccggagagagaagtgttagagtggaggttt
gacagccgcctagcatttcatcacgtggcccgagagctgcatccggagtacttcaagaactgctgatatcgagcttgctacaagg
gactttccgctggggactttccagggaggcgtggcctgggcgggactggggagtggcgagccctcagatcctgcatataagca
gctgctttttgcctgtactgggtctctctggttagaccagatctgagcctgggagctctctggctaactagggaacccactgcttaag
cctcaataaagcttgccttgagtgcttcaagtagtgtgtgcccgtctgttgtgtgactctggtaactagagatccctcagacccttta
gtcagtgtggaaaatctctagcagtggcgcccgaacagggacttgaaagcgaaagggaaaccagaggagctctctcgacgca
ggactcggcttgctgaagcgcgcacggcaagaggcgaggggcggcgactggtgagtacgccaaaaattttgactagcggag
gctagaaggagagagatgggtgcgagagcgtcagtattaagcgggggagaattagatcgcgatgggaaaaaattcggttaagg
ccagggggaaagaaaaaatataaattaaaacatatagtatgggcaagcagggagctagaacgattcgcagttaatcctggcctg
ttagaaacatcagaaggctgtagacaaatactgggacagctacaaccatcccttcagacaggatcagaagaacttagatcattat
ataatacagtagcaaccctctattgtgtgcatcaaaggatagagataaaagacaccaaggaagctttagacaagatagaggaag
agcaaaacaaaagtaagaccaccgcacagcaagcggccggtgatcttcagacctggacgatatatatgagggacaattggag
aagtgaattatataaatataaagtagtaaaaattgaaccattaggagtagcacccaccaaggcaaagagaagagtggtgcagag
agaaaaaagagcagtgggaataggagctttgttccttgggttcttgggagcagcaggaagcactatgggcgcagcgtcaatgac
gctgacggtacaggccagacaattattgtctggtatagtgcagcagcagaacaatttgctgagggctattgaggcgcaacagcat
ctgttgcaactcacagtctggggcatcaagcagctccaggcaagaatcctggctgtggaaagatacctaaaggatcaacagctc
ctggggatttggggttgctctggaaaactcatttgcaccactgctgtgccttggaatgctagttggagtaataaatctctggaacaga
tttggaatcacacgacctggatggagtgggacagagaaattaacaattacacaagcttaatacactccttaattgaagaatcgcaa
aaccagcaagaaaagaatgaacaagaattattggaattagataaatgggcaagtttgtggaattggtttaacataacaaattggct
gtggtatataaaattattcataatgatagtaggaggcttggtaggtttaagaatagtttttgctgtactttctatagtgaatagagttaggc
agggatattcaccattatcgtttcagacccacctcccaaccccgaggggacccgacaggcccgaaggaatagaagaagaagg
tggagagagagacagagacagatccattcgattagtgaacggatctcgacggtcgccaaatggcagtattcatccacaattttaa
aagaaaaggggggattggggggtacagtgcaggggaaagaatagtagacataatagcaacagacatacaaactaaagaatta
caaaaacaaattacaaaaattcaaaattttcgggtttattacagggacagcagagatccagtttggatcgataagcttgatatcgaat
tcctgcagccccgataaaataaaagattttatttagtctccagaaaaaggggggaatgaaagaccccacctgtaggtttggcaag
ctagctgcagtaacgccattttgcaaggcatggaaaaataccaaaccaagaatagagaagttcagatcaagggcgggtacatg
aaaatagctaacgttgggccaaacaggatatctgcggtgagcagtttcggccccggcccggggccaagaacagatggtcacc
gcagtttcggccccggcccgaggccaagaacagatggtccccagatatggcccaaccctcagcagtttcttaagacccatcag
atgtttccaggctcccccaaggacctgaaatgaccctgcgccttatttgaattaaccaatcagcctgcttctcgcttctgttcgcgcg
cttctgcttcccgagctctataaaagagctcacaacccctcactcggcgcgccagtcctccgacagactgagtcgcccggggg
ggatctggagctctcgagaattctcacgcgtctgcaggatatcaagcttgcggtaccgcgggcccgggatccaccggtcgccac
catggtgagcaagggcgaggaggataacatggccatcattaaggagttcatgcgcttcaaggtgcacatggagggtccgtga
acggccacgtgttcgagatcgagggcgagggcgagggccgcccctacgagggcacccagaccgccaagctgaaggtgacc
aagggtggcccccctgcccttcacctgggacatcctgtcccctcaattcatgtacggctccaatgcctacgtgaagcaccccgcc
gacatccccgactactttaagctgtccttccccgagggcttcaagtgggagcgcgtgatgaaattcgaggacggcggcgtggtg
accgtgacccaggactcctccctgcaggacggtgagttcatctacaaggtgaagctgcgcggcaccaacttcccctccgacgg

ccccgtaatgcagaagaagaccatgggctgggaggccctctccgagcggatgtaccccgaggacggcgccctgaagggcg
aggtcaagccgagagtgaagctgaaggacggcggccactacgacgctgaggtcaagaccacctacaaggccaagaagccc
gtgcagctgcccggcgcctacaacgtcaaccgcaagttggacatcacctcacacaacgaggactacaccatcgtggaacagt
acgaacgtgccgagggccgccactccaccggcggcatggacgagctgtacaagggaagtggaagcggtagcggctcaggt
agtggaagtcccaagaagaagcgcaaggtgtaaagcggccgcgactctagagtcgacctgcaggcatgcaagcttgatatca
agcttatcgataatcaacctctggattacaaaatttgtgaaagattgactggtattcttaactatgttgctccttttacgctatgtggatac
gctgctttaatgcctttgtatcatgctattgcttcccgtatggctttcattttctcctccttgtataaatcctggttgctgtctctttatgagga
gttgtggcccgttgtcaggcaacgtggcgtggtgtgcactgtgtttgctgacgcaacccccactggttggggcattgccaccacct
gtcagctcctttccgggactttcgctttccccctccctattgccacggcggaactcatcgccgcctgccttgcccgctgctggaca
ggggctcggctgttgggcactgacaattccgtggtgttgtcggggaaatcatcgtcctttccttggctgctcgcctgtgttgccacct
ggattctgcgcgggacgtccttctgctacgtcccttcggccctcaatccagcggaccttccttcccgcggcctgctgccggctctg
cggcctcttccgcgtcttcgccttcgccctcagacgagtcggatctccctttgggccgcctccccgcat

H2B-mGFP
attaattccgtgtattctatagtgtcacctaaatcgtatgtgtatgatacataaggttatgtattaattgtagccgcgttctaacgacaata
tgtacaagcctaattgtgtagcatctggcttactgaagcagaccctatcatctctctcgtaaactgccgtcagagtcggtttggttgg
acgaaccttctgagtttctggtaacgccgtcccgcacccggaaatggtcagcgaaccaatcagcagggtcatcgctagccagat
cctctacgccggacgcatcgtggccggcatcaccggcgccacaggtgcggttgctggcgcctatatcgccgacatcaccgatg
gggaagatcgggctcgccacttcgggctcatgagcgcttgtttcggcgtgggtatggtggcaggccccgtggccgggggactgt
tgggcgccatctccttgcatgcaccattccttgcggcggcggtgctcaacggcctcaacctactactgggctgcttcctaatgcag
gagtcgcataagggagagcgtcgaatggtgcactctcagtacaatctagctctgatgccgcatagttaagccagccccgacacc
cgccaacacccgctgacgcgccctgacgggcttgtctgctcccggcatccgcttacagacaagctgtgaccgtctccgggagc
tgcatgtgtcagaggttttcaccgtcatcaccgaaacgcgcgagacgaaagggcctcgtgatacgcctatttttataggttaatgtc
atgataataatggtttcttagacgtcaggtggcacttttcggggaaatgtgcgcggaacccctatttgtttatttttctaaatacattcaa
atatgtatccgctcatgagacaataaccctgataaatgcttcaataatattgaaaaaggaagagtatgagtattcaacatttccgtgt
cgcccttattcccttttttgcggcattttgccttcctgtttttgctcacccagaaacgctggtgaaagtaaaagatgctgaagatcagtt
gggtgcacgagtgggttacatcgaactggatctcaacagcggtaagatccttgagagttttcgccccgaagaacgttttccaatga
tgagcacttttaaagttctgctatgtggcgcggtattatcccgtattgacgccgggcaagagcaactcggtcgccgcatacactatt
ctcagaatgacttggttgagtactcaccagtcacagaaaagcatcttacggatggcatgacagtaagagaattatgcagtgctgcc
ataaccatgagtgataacactgcggccaacttacttctgacaacgatcggaggaccgaaggagctaaccgcttttttgcacaaca
tgggggatcatgtaactcgccttgatcgttgggaaccggagctgaatgaagccataccaaacgacgagcgtgacaccacgatg
cctgtagcaatggcaacaacgttgcgcaaactattaactggcgaactacttactctagcttcccggcaacaattaatagactggat
ggaggcggataaagttgcaggaccacttctgcgctcggcccttccggctggctggtttattgctgataaatctggagccggtgag
cgtgggtctcgcggtatcattgcagcactggggccagatggtaagccctcccgtatcgtagttatctacacgacggggagtcagg
caactatggatgaacgaaatagacagatcgctgagataggtgcctcactgattaagcattggtaactgtcagaccaagtttactcat
atatactttagattgatttaaaacttcatttttaatttaaaaggatctaggtgaagatcctttttgataatctcatgaccaaaatcccttaac
gtgagttttcgttccactgagcgtcagaccccgtagaaaagatcaaaggatcttcttgagatcctttttttctgcgcgtaatctgctgct
tgcaaacaaaaaaaccaccgctaccagcggtggtttgtttgccggatcaagagctaccaactctttttccgaaggtaactggcttc
agcagagcgcagataccaaatactgtccttctagtgtagccgtagttaggccaccacttcaagaactctgtagcaccgcctacat
acctcgctctgctaatcctgttaccagtggctgctgccagtggcgataagtcgtgtcttaccgggttggactcaagacgatagttac
cggataaggcgcagcggtcgggctgaacggggggttcgtgcacacagcccagcttggagcgaacgacctacaccgaactga
gatacctacagcgtgagctatgagaaagcgccacgcttcccgaagggagaaaggcggacaggtatccggtaagcggcaggg
tcggaacaggagagcgcacgagggagcttccagggggaaacgcctggtatctttatagtcctgtcgggtttcgccacctctgact
tgagcgtcgatttttgtgatgctcgtcaggggggcggagcctatggaaaaacgccagcaacgcggcctttttacggttcctggcct
tttgctggccttttgctcacatgttctttcctgcgttatcccctgattctgtggataaccgtattaccgcctttgagtgagctgataccgct
cgccgcagccgaacgaccgagcgcagcgagtcagtgagcgaggaagcggaagagcgcccaatacgcaaaccgcctctcc
ccgcgcgttggccgattcattaatgcagctgtggaatgtgtgtcagttagggtgtggaaagtccccaggctccccagcaggcaga
agtatgcaaagcatgcatctcaattagtcagcaaccaggtgtggaaagtccccaggctccccagcaggcagaagtatgcaaag
catgcatctcaattagtcagcaaccatagtcccgcccctaactccgcccatcccgcccctaactccgcccagttccgcccattct
ccgccccatggctgactaattttttttatttatgcagaggccgaggccgcctcggcctctgagctattccagaagtagtgaggaggc
ttttttggaggcctaggcttttgcaaaaagcttggacacaagacaggcttgcgagatatgtttgagaataccactttatcccgcgtca
gggagaggcagtgcgtaaaaagacgcggactcatgtgaaatactggtttttagtgcgccagatctctataatctcgcgcaacctat
tttcccctcgaacactttttaagccgtagataaacaggctgggacacttcacatgagcgaaaaatacatcgtcacctgggacatgtt
gcagatccatgcacgtaaactcgcaagccgactgatgccttctgaacaatggaaaggcattattgccgtaagccgtggcggtctg

taccgggtgcgttactggcgcgtgaactgggtattcgtcatgtcgataccgtttgtatttccagctacgatcacgacaaccagcgcg
agcttaaagtgctgaaacgcgcagaaggcgatggcgaaggcttcatcgttattgatgacctggtggataccggtggtactgcggt
tgcgattcgtgaaatgtatccaaaagcgcactttgtcaccatcttcgcaaaaccggctggtcgtccgctggttgatgactatgttgtt
gatatcccgcaagatacctggattgaacagccgtgggatatgggcgtcgtattcgtcccgccaatctccggtcgctaatcttttcaa
cgcctggcactgccgggcgttgttctttttaacttcaggcgggttacaatagtttccagtaagtattctggaggctgcatccatgaca
caggcaaacctgagcgaaaccctgttcaaaccccgctttaaacatcctgaaacctcgacgctagtccgccgctttaatcacggc
gcacaaccgcctgtgcagtcggcccttgatggtaaaaccatccctcactggtatcgcatgattaaccgtctgatgtggatctggcg
cggcattgacccacgcgaaatcctcgacgtccaggcacgtattgtgatgagcgatgccgaacgtaccgacgatgatttatacgat
acggtgattggctaccgtggcggcaactggatttatgagtgggccccggatctttgtgaaggaaccttacttctgtggtgtgacata
attggacaaactacctacagagatttaaagctctaaggtaaatataaaatttttaagtgtataatgtgttaaactactgattctaattgttt
gtgtattttagattccaacctatggaactgatgaatgggagcagtggtggaatgcctttaatgaggaaaacctgttttgctcagaaga
aatgccatctagtgatgatgaggctactgctgactctcaacattctactcctccaaaaaagaagagaaaggtagaagaccccaag
gactttccttcagaattgctaagttttttgagtcatgctgtgtttagtaatagaactcttgcttgctttgctatttacaccacaaaggaaaa
agctgcactgctatacaagaaaattatggaaaaatattctgtaacctttataagtaggcataacagttataatcataacatactgtttttt
cttactccacacaggcatagagtgtctgctattaataactatgctcaaaaattgtgtacctttagctttttaatttgtaaaggggttaata
aggaatatttgatgtatagtgccttgactagagatcataatcagccataccacatttgtagaggttttacttgctttaaaaaacctccca
cacctccccctgaacctgaaacataaaatgaatgcaattgttgttgttaacttgtttattgcagcttataatggttacaaataaagcaat
agcatcacaaatttcacaaataaagcatttttttcactgcattctagttgtggtttgtccaaactcatcaatgtatcttatcatgtctggat
caactggataactcaagctaaccaaaatcatcccaaacttcccaccccataccctattaccactgccaattacctagtggtttcattt
actctaaacctgtgattcctctgaattattttcattttaaagaaattgtatttgttaaatatgtactacaaacttagtagttggaagggctaa
ttcactcccaaagaagacaagatatccttgatctgtggatctaccacacacaaggctacttccctgattagcagaactacacacca
gggccaggggtcagatatccactgacctttggatggtgctacaagctagtaccagttgagccagataaggtagaagaggccaat
aaaggagagaacaccagcttgttacaccctgtgagcctgcatgggatggatgacccggagagagaagtgttagagtggaggttt
gacagccgcctagcatttcatcacgtggcccgagagctgcatccggagtacttcaagaactgctgatatcgagcttgctacaagg
gactttccgctggggactttccagggaggcgtggcctgggcgggactggggagtggcgagccctcagatcctgcatataagca
gctgctttttgcctgtactgggtctctctggttagaccagatctgagcctgggagctctctggctaactagggaacccactgcttaag
cctcaataaagcttgccttgagtgcttcaagtagtgtgtgcccgtctgttgtgtgactctggtaactagagatccctcagaccctttta
gtcagtgtggaaaatctctagcagtggcgcccgaacagggacttgaaagcgaaagggaaaccagaggagctctctcgacgca
ggactcggcttgctgaagcgcgcacggcaagaggcgaggggcggcgactggtgagtacgccaaaaattttgactagcggag
gctagaaggagagagatgggtgcgagagcgtcagtattaagcgggggagaattagatcgcgatgggaaaaaattcggttaagg
ccagggggaaagaaaaaatataaattaaaacatatagtatgggcaagcagggagctagaacgattcgcagttaatcctggcctg
ttagaaacatcagaaggctgtagacaaatactgggacagctacaaccatcccttcagacaggatcagaagaacttagatcattat
ataatacagtagcaaccctctattgtgtgcatcaaaggatagagataaaagacaccaaggaagctttagacaagatagaggaag
agcaaaacaaaagtaagaccaccgcacagcaagcggccggtgatcttcagacctggacgatatatatgagggacaattggag
aagtgaattatataaatataaagtagtaaaaattgaaccattaggagtagcacccaccaaggcaaagagaagagtggtgcagag
agaaaaaagagcagtgggaataggagctttgttccttgggttcttgggagcagcaggaagcactatgggcgcagcgtcaatgac
gctgacggtacaggccagacaattattgtctggtatagtgcagcagcagaacaatttgctgagggctattgaggcgcaacagcat
ctgttgcaactcacagtctggggcatcaagcagctccaggcaagaatcctggctgtggaaagatacctaaaggatcaacagctc
ctggggatttggggttgctctggaaaactcatttgcaccactgctgtgccttggaatgctagttggagtaataaatctctggaacaga
tttggaatcacacgacctggatggagtgggacagagaaattaacaattacacaagcttaatacactccttaattgaagaatcgcaa
aaccagcaagaaaagaatgaacaagaattattggaattagataaatgggcaagtttgtggaattggtttaacataacaaattggct
gtggtatataaaattattcataatgatagtaggaggcttggtaggtttaagaatagtttttgctgtactttctatagtgaatagagttaggc
agggatattcaccattatcgtttcagacccacctcccaaccccgaggggacccgacaggcccgaaggaatagaagaagaagg
tggagagagagacagagacagatccattcgattagtgaacggatctcgacggtATcgccaaatggcagtattcatccacaatttt
aaaagaaaaggggggattggggggtacagtgcaggggaaagaatagtagacataatagcaacagacatacaaactaaagaat
tacaaaaacaaattacaaaaattcaaaattttcgggtttattacagggacagcagagatccagtttggatcgataagcttgatatcga
attcctgcagcccgataaaataaaagattttatttagtctccagaaaaaggggggaatgaaagaccccacctgtaggtttggcaa
gctagctgcagtaacgccattttgcaaggcatggaaaaataccaaaccaagaatagagaagttcagatcaagggcgggtacat
gaaaatagctaacgttgggccaaacaggatatctgcggtgagcagtttcggccccggcccggggccaagaacagatggtcac
cgcagtttcggccccggcccgaggccaagaacagatggtccccagatatggcccaaccctcagcagtttcttaagacccatca
gatgtttccaggctcccccaaggacctgaaatgaccctgcgccttatttgaattaaccaatcagcctgcttctcgcttctgttcgcgc
gcttctgcttcccgagctctataaaagagctcacaacccctcactcggcgcgccagtcctccgacagactgagtcgcccgggg
gggatctggagctctcgagaattctcACGCGTGCCGGTACCATGTTGGATCCAATGCCTGAGCCAGCCA
AGTCTGCTCCCGCCCCGAAGAAGGGCTCCAAGAAGGCAGTGACCAAAGCGCAGAAGAAAGA

TGGCAAGAAGCGCAAGCGCAGCCGCAAGGAGAGTTACTCTGTGTACGTGTACAAGGTGCTGA
AACAGGTCCATCCCGACACTGGCATCTCTTCCAAGGCCATGGGCATCATGAATTCTTTCGTTAA
CGACATATTTGAGCGCATCGCGGGCGAGGCTTCCCGCCTGGCGCATTACAACAAGCGCTCGA
CCATCACCTCCAGGGAGATCCAGACGGCCGTGCGCCTGCTGCTTCCCGGAGAGCTGGCCAA
GCACGCCGTGTCGGAGGGCACCAAGGCCGTCACCAAGTACACCAGCTCCAAGGGTGGAGG
TGGAAGCGGTgtgagcaagggcgaggagctgttcaccggggtggtgcccatcctggtcgagctggacggcgacgtaaa
cggccacaagttcagcgtgtccggcgagggcgagggcgatgccacctacggcaagctgaccctgaagttcatctgcaccacc
ggcaagctgcccgtgccctggcccaccctcgtgaccaccctgacctacggcgtgcagtgcttcagccgctaccccgaccacat
gaagcagcacgacttcttcaagtccgccatgcccgaaggctacgtccaggagcgcaccatcttcttcaaggacgacggcaact
acaagacccgcgccgaggtgaagttcgagggcgacaccctggtgaaccgcatcgagctgaagggcatcgacttcaaggagg
acggcaacatcctggggcacaagctggagtacaactacaacagccacaacgtctatatcatggccgacaagcagaagaacg
gcatcaaggtgaacttcaagatccgccacaacatcgaggacggcagcgtgcagctcgccgaccactaccagcagaacaccc
ccatcggcgacggccccgtgctgctgcccgacaaccactacctgagcacccagtccaagctgagcaaagaccccaacgaga
agcgcgatcacatggtcctgctggagttcgtgaccgccgccgggatcactctcggcatggacgagctgtacaagGGTGGAG
GTCGGACCGAAGAGTACAAGCTTATCCTGAACGGTAAAACCCTGAAAGGTGAAACCACCACC
GAAGCTGTTGACGCTGCTACCGCGGAAAAAGTTTTCAAACAGTACGCTAACGACAACGGTGTT
GACGGTGAATGGACCTACGACGACGCTACCAAAACCTTCACGGTAACCGAATAAACTAGTTAA
GCGGCCGCgactctagagtcgaccctttaagaccaatgacttacaaggcagctgtagatcttagccactttttaaaagaaaag
gggggactggaagggctaattcactcccaacgaagacaagatctgctttttgcttgtactgggtctctctggttagaccagatctga
gcctgggagctctctggctaactagggaacccactgcttaagcctcaataaagcttgccttgagtgcttcaagtagtgtgtgcccgt
ctgttgtgtgactctggtaactagagatccctcagacccttttagtcagtgtggaaaatctctagcagcatctaga

mIFP-NLS
attaattccgtgtattctatagtgtcacctaaatcgtatgtgtatgatacataaggttatgtattaattgtagccgcgttctaacgacaata
tgtacaagcctaattgtgtagcatctggcttactgaagcagaccctatcatctctctcgtaaactgccgtcagagtcggtttggttgg
acgaaccttctgagtttctggtaacgccgtcccgcacccggaaatggtcagcgaaccaatcagcagggtcatcgctagccagat
cctctacgccggacgcatcgtggccggcatcaccggcgccacaggtgcggttgctggcgcctatatcgccgacatcaccgatg
gggaagatcgggctcgccacttcgggctcatgagcgcttgtttcggcgtgggtatggtggcaggccccgtggccgggggactgt
tgggcgccatctccttgcatgcaccattccttgcggcggcggtgctcaacggcctcaacctactactgggctgcttcctaatgcag
gagtcgcataagggagagcgtcgaatggtgcactctcagtacaatctagctctgatgccgcatagttaagccagccccgacacc
cgccaacacccgctgacgcgccctgacgggcttgtctgctcccggcatccgcttacagacaagctgtgaccgtctccgggagc
tgcatgtgtcagaggttttcaccgtcatcaccgaaacgcgcgagacgaaagggcctcgtgatacgcctatttttataggttaatgtc
atgataataatggtttcttagacgtcaggtggcacttttcggggaaatgtgcgcggaacccctatttgtttatttttctaaatacattcaa
atatgtatccgctcatgagacaataaccctgataaatgcttcaataatattgaaaaaggaagagtatgagtattcaacatttccgtgt
cgcccttattcccttttttgcggcattttgccttcctgtttttgctcacccagaaacgctggtgaaagtaaaagatgctgaagatcagtt
gggtgcacgagtgggttacatcgaactggatctcaacagcggtaagatccttgagagttttcgccccgaagaacgttttccaatga
tgagcacttttaaagttctgctatgtggcgcggtattatcccgtattgacgccgggcaagagcaactcggtcgccgcatacactatt
ctcagaatgacttggttgagtactcaccagtcacagaaaagcatcttacggatggcatgacagtaagagaattatgcagtgctgcc
ataaccatgagtgataacactgcggccaacttacttctgacaacgatcggaggaccgaaggagctaaccgcttttttgcacaaca
tgggggatcatgtaactcgccttgatcgttgggaaccggagctgaatgaagccataccaaacgacgagcgtgacaccacgatg
cctgtagcaatggcaacaacgttgcgcaaactattaactggcgaactacttactctagcttcccggcaacaattaatagactggat
ggaggcggataaagttgcaggaccacttctgcgctcggcccttccggctggctggtttattgctgataaatctggagccggtgag
cgtgggtctcgcggtatcattgcagcactggggccagatggtaagccctcccgtatcgtagttatctacacgacggggagtcagg
caactatggatgaacgaaatagacagatcgctgagataggtgcctcactgattaagcattggtaactgtcagaccaagtttactcat
atatactttagattgatttaaaacttcatttttaatttaaaaggatctaggtgaagatcctttttgataatctcatgaccaaaatcccttaac
gtgagttttcgttccactgagcgtcagaccccgtagaaaagatcaaaggatcttcttgagatcctttttttctgcgcgtaatctgctgct
tgcaaacaaaaaaaccaccgctaccagcggtggtttgtttgccggatcaagagctaccaactctttttccgaaggtaactggcttc
agcagagcgcagataccaaatactgtccttctagtgtagccgtagttaggccaccacttcaagaactctgtagcaccgcctacat
acctcgctctgctaatcctgttaccagtggctgctgccagtggcgataagtcgtgtcttaccgggttggactcaagacgatagttac
cggataaggcgcagcggtcgggctgaacggggggttcgtgcacacagcccagcttggagcgaacgacctacaccgaactga
gatacctacagcgtgagctatgagaaagcgccacgcttcccgaagggagaaaggcggacaggtatccggtaagcggcaggg
tcggaacaggagagcgcacgagggagcttccagggggaaacgcctggtatctttatagtcctgtcgggtttcgccacctctgact
tgagcgtcgatttttgtgatgctcgtcaggggggcggagcctatggaaaaacgccagcaacgcggcctttttacggttcctggcct
tttgctggccttttgctcacatgttctttcctgcgttatcccctgattctgtggataaccgtattaccgcctttgagtgagctgataccgct

cgccgcagccgaacgaccgagcgcagcgagtcagtgagcgaggaagcggaagagcgcccaatacgcaaaccgcctctcc
ccgcgcgttggccgattcattaatgcagctgtggaatgtgtgtcagttagggtgtggaaagtccccaggctccccagcaggcaga
agtatgcaaagcatgcatctcaattagtcagcaaccaggtgtggaaagtccccaggctccccagcaggcagaagtatgcaaag
catgcatctcaattagtcagcaaccatagtcccgcccctaactccgcccatcccgcccctaactccgcccagttccgcccattct
ccgccccatggctgactaattttttttatttatgcagaggccgaggccgcctcggcctctgagctattccagaagtagtgaggaggc
ttttttggaggcctaggcttttgcaaaaagcttggacacaagacaggcttgcgagatatgtttgagaataccactttatcccgcgtca
gggagaggcagtgcgtaaaaagacgcggactcatgtgaaatactggtttttagtgcgccagatctctataatctcgcgcaacctat
tttcccctcgaacactttttaagccgtagataaacaggctgggacacttcacatgagcgaaaaatacatcgtcacctgggacatgtt
gcagatccatgcacgtaaactcgcaagccgactgatgccttctgaacaatggaaaggcattattgccgtaagccgtggcggtctg
taccgggtgcgttactggcgcgtgaactgggtattcgtcatgtcgataccgtttgtatttccagctacgatcacgacaaccagcgcg
agcttaaagtgctgaaacgcgcagaaggcgatggcgaaggcttcatcgttattgatgacctggtggataccggtggtactgcggt
tgcgattcgtgaaatgtatccaaaagcgcactttgtcaccatcttcgcaaaaccggctggtcgtccgctggttgatgactatgttgtt
gatatcccgcaagatacctggattgaacagccgtgggatatgggcgtcgtattcgtcccgccaatctccggtcgctaatcttttcaa
cgcctggcactgccgggcgttgttctttttaacttcaggcgggttacaatagtttccagtaagtattctggaggctgcatccatgaca
caggcaaacctgagcgaaaccctgttcaaaccccgctttaaacatcctgaaacctcgacgctagtccgccgctttaatcacggc
gcacaaccgcctgtgcagtcggcccttgatggtaaaaccatccctcactggtatcgcatgattaaccgtctgatgtggatctggcg
cggcattgacccacgcgaaatcctcgacgtccaggcacgtattgtgatgagcgatgccgaacgtaccgacgatgatttatacgat
acggtgattggctaccgtggcggcaactggatttatgagtgggccccggatctttgtgaaggaaccttacttctgtggtgtgacata
attggacaaactacctacagagatttaaagctctaaggtaaatataaaatttttaagtgtataatgtgttaaactactgattctaattgttt
gtgtattttagattccaacctatggaactgatgaatgggagcagtggtggaatgcctttaatgaggaaaacctgttttgctcagaaga
aatgccatctagtgatgatgaggctactgctgactctcaacattctactcctccaaaaaagaagagaaaggtagaagaccccaag
gactttccttcagaattgctaagttttttgagtcatgctgtgtttagtaatagaactcttgcttgctttgctatttacaccacaaaggaaaa
agctgcactgctatacaagaaaattatggaaaaatattctgtaacctttataagtaggcataacagttataatcataacatactgtttttt
cttactccacacaggcatagagtgtctgctattaataactatgctcaaaaattgtgtacctttagctttttaatttgtaaaggggttaata
aggaatatttgatgtatagtgccttgactagagatcataatcagccataccacatttgtagaggttttacttgctttaaaaaacctccca
cacctccccctgaacctgaaacataaaatgaatgcaattgttgttgttaacttgtttattgcagcttataatggttacaaataaagcaat
agcatcacaaatttcacaaataaagcatttttttcactgcattctagttgtggtttgtccaaactcatcaatgtatcttatcatgtctggat
caactggataactcaagctaaccaaaatcatcccaaacttcccaccccataccctattaccactgccaattacctagtggtttcattt
actctaaacctgtgattcctctgaattattttcattttaaagaaattgtatttgttaaatatgtactacaaacttagtagttggaagggctaa
ttcactcccaaagaagacaagatatccttgatctgtggatctaccacacacaaggctacttccctgattagcagaactacacacca
gggccaggggtcagatatccactgacctttggatggtgctacaagctagtaccagttgagccagataaggtagaagaggccaat
aaaggagagaacaccagcttgttacaccctgtgagcctgcatgggatggatgacccggagagagaagtgttagagtggaggttt
gacagccgcctagcatttcatcacgtggcccgagagctgcatccggagtacttcaagaactgctgatatcgagcttgctacaagg
gactttccgctggggactttccagggaggcgtggcctgggcgggactggggagtggcgagccctcagatcctgcatataagca
gctgctttttgcctgtactgggtctctctggttagaccagatctgagcctgggagctctctggctaactagggaacccactgcttaag
cctcaataaagcttgccttgagtgcttcaagtagtgtgtgcccgtctgttgtgtgactctggtaactagagatccctcagacccttta
gtcagtgtggaaaatctctagcagtggcgcccgaacagggacttgaaagcgaaagggaaaccagaggagctctctcgacgca
ggactcggcttgctgaagcgcgcacggcaagaggcgaggggcggcgactggtgagtacgccaaaaattttgactagcggag
gctagaaggagagagatgggtgcgagagcgtcagtattaagcgggggagaattagatcgcgatgggaaaaaattcggttaagg
ccagggggaaagaaaaaatataaattaaaacatatagtatgggcaagcagggagctagaacgattcgcagttaatcctggcctg
ttagaaacatcagaaggctgtagacaaatactgggacagctacaaccatcccttcagacaggatcagaagaacttagatcattat
ataatacagtagcaaccctctattgtgtgcatcaaaggatagagataaaagacaccaaggaagctttagacaagatagaggaag
agcaaaacaaaagtaagaccaccgcacagcaagcggccggtgatcttcagacctggacgatatatatgagggacaattggag
aagtgaattatataaatataaagtagtaaaaattgaaccattaggagtagcacccaccaaggcaaagagaagagtggtgcagag
agaaaaaagagcagtgggaataggagctttgttccttgggttcttgggagcagcaggaagcactatgggcgcagcgtcaatgac
gctgacggtacaggccagacaattattgtctggtatagtgcagcagcagaacaatttgctgagggctattgaggcgcaacagcat
ctgttgcaactcacagtctggggcatcaagcagctccaggcaagaatcctggctgtggaaagatacctaaaggatcaacagctc
ctggggatttggggttgctctggaaaactcatttgcaccactgctgtgccttggaatgctagttggagtaataaatctctggaacaga
tttggaatcacacgacctggatggagtgggacagagaaattaacaattacacaagcttaatacactccttaattgaagaatcgcaa
aaccagcaagaaaagaatgaacaagaattattggaattagataaatgggcaagtttgtggaattggtttaacataacaaattggct
gtggtatataaaattattcataatgatagtaggaggcttggtaggtttaagaatagtttttgctgtactttctatagtgaatagagttaggc
agggatattcaccattatcgtttcagacccacctcccaaccccgaggggacccgacaggcccgaaggaatagaagaagaagg
tggagagagagacagagacagatccattcgattagtgaacggatctcgacggtATcgccaaatggcagtattcatccacaatttt
aaaagaaaaggggggattggggggtacagtgcaggggaaagaatagtagacataatagcaacagacatacaaactaaagaat

tacaaaaacaaattacaaaaattcaaaattttcgggtttattacagggacagcagagatccagtttggatcgataagcttgatatcga
attcctgcagcccgataaaataaaagattttatttagtctccagaaaaaggggggaatgaaagaccccacctgtaggtttggcaa
gctagctgcagtaacgccattttgcaaggcatggaaaaataccaaaccaagaatagagaagttcagatcaagggcgggtacat
gaaaatagctaacgttgggccaaacaggatatctgcggtgagcagtttcggccccggcccggggccaagaacagatggtcac
cgcagtttcggccccggcccgaggccaagaacagatggtccccagatatggcccaaccctcagcagtttcttaagacccatca
gatgtttccaggctcccccaaggacctgaaatgaccctgcgccttatttgaattaaccaatcagcctgcttctcgcttctgttcgcgc
gcttctgcttcccgagctctataaaagagctcacaacccctcactcggcgcgccagtcctccgacagactgagtcgcccgggg
gggatctggagctctcgagaattctcACGCGTGCCGGTACCATGTCGGTACCGCTGACTACCTCAGCATT
CGGCCACGCGTTTCTGGCTAACTGTGAACGCGAGCAGATCCACCTGGCGGGCTCCATTCAG
CCGCACGGTATCCTGCTGGCTGTGAAAGAGCCGGACAACGTGGTGATCCAGGCTTCTATTAAC
GCTGCGGAGTTCCTGAACACCAACTCTGTTGTTGGCCGTCCGCTGCGTGACCTGGGCGGCG
ATCTGCCTTTGCAGATCCTGCCGCACCTGAACGGCCCGCTGCACCTGGCTCCGATGACCCTG
CGTTGTACCGTGGGTTCTCCGCCGCGTCGTGTGGACTGTACCATTCATCGTCCGTCTAACGGC
GGCCTGATCGTAGAACTGGAACCAGCAACCAAGACCACTAACATTGCGCCGGCTCTGGACGG
TGCGTTTCATCGTATCACTTCTTCATCCTCCCTGATGGGCCTGTGTGACGAAACCGCGACTATT
ATCCGTGAGATTACTGGCTACGACCGTGTGATGGTAGTACGTTTCGATGAAGAGGGTAATGGCG
AAATTCTGTCCGAACGTCGTCGTGCGGACCTGGAAGCGTTCCTGGGTAACCGCTACCCGGCG
TCTACTATTCCGCAGATCGCTCGTCGCCTGTACGAACATAACCGTGTTCGCCTGCTGGTAGATG
TGAACTATACTCCGGTTCCGCTACAGCCGCGCATCAGCCCGCTGAACGGTCGTGATCTGGATA
TGTCCCTGTCTTGCCTGCGCTCTATGTCCCCGATCCACCAGAAATACATGCAGGACATGGGCG
TTGGCGCGACCCTGGTTTGCTCTCTGATGGTGTCTGGTCGTCTGTGGGGTCTGATCGCTTGC
CACCACTACGAACCGCGCTTCGTTCCGTTCCACATTCGCGCTGCTGGCGAAGCGCTGGCGG
AAACTTGTGCGATCCGCATCGCGACGCTGGAGAGCTTTGCACAGTCTCAGTCCAAAGGTGGA
GGTCGGACCGAAGAGTACAAGCTTATCCTGAACGGTAAAACCCTGAAAGGTGAAACCACCAC
CGAAGCTGTTGACGCTGCTACCGCGGAAAAAGTTTTCAAACAGTACGCTAACGACAACGGTGT
TGACGGTGAATGGACCTACGACGACGCTACCAAAACCTTCACGGTAACCGAAGGTGGTGGTA
GCGGTGGTGGTACTAGTCCAAAAACAAGGAGGAGACCGCGAAGATCACAACGGAAAAGGCC
GCCTACGCCATGGCCGCATATGTAAGCGGCCGCgactctagagtcgaccctttaagaccaatgacttacaagg
cagctgtagatcttagccactttttaaaagaaaaggggggactggaagggctaattcactcccaacgaagacaagatctgcttttt
gcttgtactgggtctctctggttagaccagatctgagcctgggagctctctggctaactagggaacccactgcttaagcctcaataa
agcttgccttgagtgcttcaagtagtgtgtgcccgtctgttgtgtgactctggtaactagagatccctcagacccttttagtcagtgtg
gaaaatctctagcagcatctaga
