# Supplementary material for: High-content imaging-based pooled CRISPR screens in mammalian cells
Source: J Cell Biol. 2021 Jan 19;220(2):e202008158. doi: 10.1083/jcb.202008158 (PMC7821101; doi:10.1083/jcb.202008158)
Supplement: Data S5 — shows an example of detailed bioinformatic analysis steps for analyzing nuclear screen data. [file JCB_202008158_DataS5.pdf]

Supplementary file 9

Bioinformatic analysis example

(used in nuclear size screen, H2B-mGFP screen and FSC screen)

To explain the analysis steps in detail, we use the first replicate of the nuclear size screen as an example.

I. Data filtering

sgRNAs with counts less than 50 were discarded

II. Two features to evaluate CRISPR-induced phenotypes

1. Severity of the phenotype (phenotypic score calculation)

The phenotypic score (ε) of each sgRNA was quantified as previously defined[1]. Specifically in our screen,

$$\epsilon_X = ln\frac{N_X^S/N_T^S}{N_X^U/N_T^U}$$

ϵ_X phenotypic score of sgRNA X

N_X^S counts of sgRNA X under selection pressure S (sorted sample)

N_T^S total counts (T) under selection pressure S (sorted sample)

N_X^U counts of sgRNA X without selection (control sample)

N_T^U total counts (T) without selection (control sample)

A phenotypic score was calculated for each sgRNA in each run. For example, after filtering, 10 sgRNAs remained that target CASP8AP2 and this is a summary of their phenotypic scores.

sgRNA	gene	phenotypicScore_run1	phenotypicScore_run2	phenotypicScore_run3	phenotypicScore_run4
CASP8AP2_+_90539843.23-P1P2	CASP8AP2	1.048103	5.235842	1.648083	1.814958
CASP8AP2_+_90539869.23-P1P2	CASP8AP2	1.224357	5.070604	1.547882	1.142335
CASP8AP2_+_90539878.23-P1P2	CASP8AP2	0.220483	-0.512036	0.369308	5.149468
CASP8AP2_-_90539614.23-P1P2	CASP8AP2	1.962319	7.268581	3.685848	3.715603
CASP8AP2_-_90539622.23-P1P2	CASP8AP2	2.240901	5.917938	1.163832	1.284903
CASP8AP2_-_90539629.23-P1P2	CASP8AP2	2.211076	1.874859	2.750140	2.417832
CASP8AP2_-_90539639.23-P1P2	CASP8AP2	2.925306	8.597521	2.291789	2.356102
CASP8AP2_-_90539644.23-P1P2	CASP8AP2	0.740974	0.449342	0.874709	0.687373
CASP8AP2_-_90539669.23-P1P2	CASP8AP2	9.511693	2.318317	3.351519	3.348469
CASP8AP2_-_90539684.23-P1P2	CASP8AP2	6.486149	2.106880	2.334971	2.537374

The phenotypic score of each sgRNA was the average of the phenotypic scores of all runs.

sgRNA	gene	phenotypicScore
CASP8AP2_+_90539843.23-P1P2	CASP8AP2	2.436746
CASP8AP2_+_90539869.23-P1P2	CASP8AP2	2.246295
CASP8AP2_+_90539878.23-P1P2	CASP8AP2	1.306806
CASP8AP2_-_90539614.23-P1P2	CASP8AP2	4.158088
CASP8AP2_-_90539622.23-P1P2	CASP8AP2	2.651894
CASP8AP2_-_90539629.23-P1P2	CASP8AP2	2.313477
CASP8AP2_-_90539639.23-P1P2	CASP8AP2	4.042680
CASP8AP2_-_90539644.23-P1P2	CASP8AP2	0.688100
CASP8AP2_-_90539669.23-P1P2	CASP8AP2	4.632499
CASP8AP2_-_90539684.23-P1P2	CASP8AP2	3.366344

sgRNAs were then clustered by transcription start sites (TSS) and the phenotypic score of a gene was calculated as the averaged phenotypic scores of all sgRNAs targeting the same TSS. For CASP8AP2, there is only one TSS and a single phenotypic score was calculated for CASP8AP2.

gene	transcripts	phenotypicScore
CASP8AP2	P1P2	2.784293

For genes like TACC3, which have more than one TSS (each TSS has 10 designed sgRNAs), phenotypic scores were calculated for each transcript.

gene	transcripts	phenotypicScore
TACC3	P1	0.464388
TACC3	P2	2.398716

2. Trustworthiness of the phenotype (p-value calculation)

The Mann-Whitney U test p-value was used to evaluate the trustworthiness of the phenotype. p-value was calculated against the non-targeting sgRNA control set. Non-targeting sgRNA are sgRNAs that have no targeting sites in the human genome and 22 were included as controls in the library.

gene	transcripts	p-value
CASP8AP2	P1P2	0.000592

With the phenotypic scores and p-values calculated, we can plot a volcano plot (phenotypic score vs -ln(p-value)) for all genes:

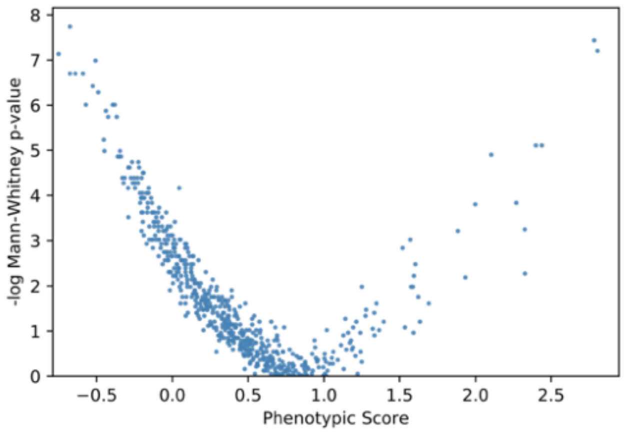

III. Hit calling

Hits were called by comparing gene behavior with background level. To determine the background “hit” rate, one could in principle determine the phenotypic scores generated by many non-targeting sgRNAs and identify true hits based on this background threshold. However, to limit library size, only 22 non-targeting sgRNAs were included in our screen, which is an insufficient sample size for this approach. Therefore, as a surrogate for non-targeting sgRNAs, we generated a collection of simulated negative controls by randomly reassigning sgRNAs in our library into groups of 10 for comparison with the genes in our screen, which consist of a group of 10 sgRNAs targeting a specific gene. Phenotypic scores for these simulated negative controls were calculated as described above. Since the hit rate of our screen is relatively low, it is unlikely that one of our simulated negative controls will have a high phenotypic score and low p-value. Thus, phenotypic scores and p-values calculated from these simulated negative controls can effectively represent the background level (negatives) of our screen. This process can be repeated to generate multiple sets of simulated negative controls as shown below (2 example sets of simulated negative controls for this screen).

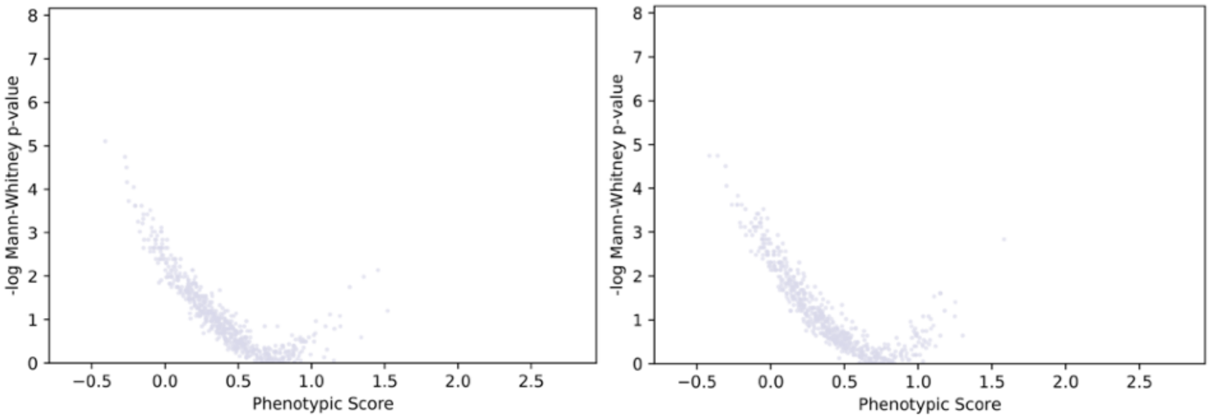

A score η summarizing effects from both severity of the phenotype (phenotypic score) as well as the confidence level (-ln(p value)) was calculated for each gene and simulated negative control:

$$\eta = (\epsilon_X + \delta) \times (-ln p_X)$$

ϵ_X phenotypic score of gene X

δ displacement applied to center the no effect phenotypic score to zero
p_X Mann-Whitney p-value of gene X

For this screen, δ is -0.8.

For each threshold of score η, we identify the number of simulated negative controls that fall above the threshold (N_C, which represent false positives) and the number of genes that fall above the threshold (N_G, the true positives). The empirical false discovery rate (eFDR) can then be calculated for a given set of simulated negative controls as shown below.

$$eFDR = \frac{N_C}{N_C + N_G} \times 100\%$$

N_C number of control groups above threshold
N_G number of genes above threshold

By repeating this with 100 different sets of simulated negative controls, an average eFDR was calculated.

Using this approach, we lowered the thresholds for score η stepwise to generate a series of eFDRs (left figure shown below). An eFDR of 0.1% was chosen as the cut-off to call hits. For this screen, the threshold of score η is 1.004.

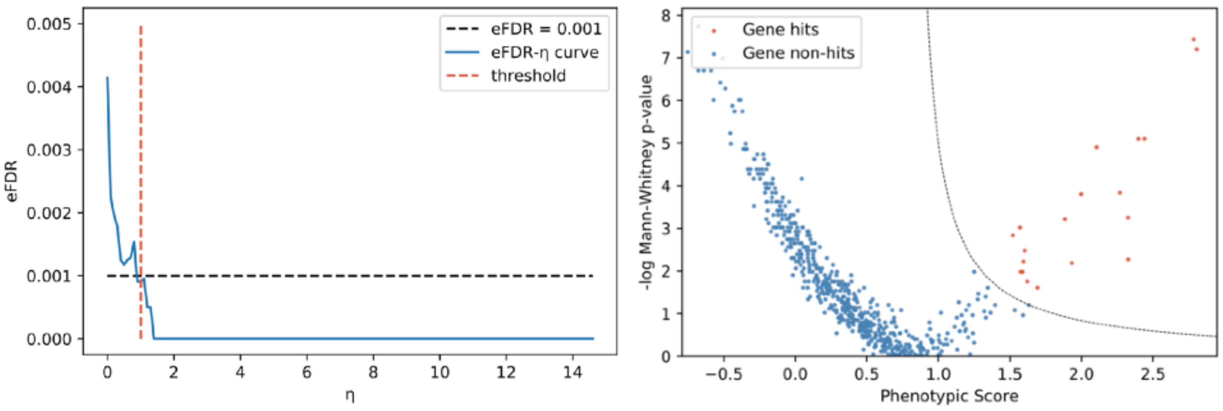

Reference

1 Kampmann, M., Bassik, M. C. & Weissman, J. S. Integrated platform for genome-wide screening and construction of high-density genetic interaction maps in mammalian cells. *Proc Natl Acad Sci U S A* **110**, E2317-2326, doi:10.1073/pnas.1307002110 (2013).
