# Supplementary material for: High-content imaging-based pooled CRISPR screens in mammalian cells
Source: J Cell Biol. 2021 Jan 19;220(2):e202008158. doi: 10.1083/jcb.202008158 (PMC7821101; doi:10.1083/jcb.202008158)
Supplement: Data S8 — lists primers used for RT-qPCR experiments. [file JCB_202008158_DataS8.pdf]

Supplementary file 12: qPCR primers used in the study

Targeted gene	Forward primer	Reverse primer
**CASP8AP2**	CACTTGCCACTTCTACAAGTC	TGGCGGCTAAATATGCAAATG
**AURKB**	TGGAAACGTGTACTTGGCTC	AGGATGTTGGGATGGTGC
**RAD51**	GTGGTAGCTCAAGTGGATGG	GGGAGAGTCGTAGATTTTGCAG
**TACC3**	CCTCTTCAAGCGTTTTGAGAAAC	GCCCTCCTGGGTGATCCTT
**INCENP**	GAGAGGCTCCTGAATGTTGAG	AATCTCCGTGTCATTGTGGG
**TICRR**	AGCAGGTGATGGAGAAGTTG	AAACAGTCCAGTATCCAAGGTG
**TOP2A**	CCTTTGCCAATGCTTCCAAGTTAC	GTGTCTTCTCGGTGCCATTCAAC
**CDCA8**	TTGACTACTTCGCCCTTG	CTTCTTCTTCCTCTTCCACTA
**FBXO5**	GTGTCTAAAGTGAGCACAACTTG	TTCTCTGGTTGAAGCATGAGG
**KIF11**	TATTGAATGGGCGCTAGCTT	TCGTCTGCGAAGAAGAAAGA
**SPDL1**	GTTTGAGCTGCGAATGTGAAG	GCCTGGCTTCATCTAATTCCAC
**SKA1**	AGTTGAAGAACCTGAACCCG	GGTTAAGCGGGATTTCATGTAC
**DNA2**	GCCGCTGGAACTTAAAACTG	GTACATCTGACCAGTCTTGAGG
**NUP62**	CTGAATCAAGGTCCAGAGAAGG	GTGCCTCCAAAATTAAACCCG
**KRI1**	AAGCCTTGGAGAAGCAGAAG	CTGGAGTCTGTGATTGGAAGTC
**ACTB**	GCTACGAGCTGCCTGACG	GGCTGGAAGAGTGCCTCA
